# Mechanical and Hygroscopic Properties of Molded Pulp Products Using Different Wood-Based Cellulose Fibers

**DOI:** 10.3390/polym13193225

**Published:** 2021-09-23

**Authors:** Claire Dislaire, Bastien Seantier, Marion Muzy, Yves Grohens

**Affiliations:** 1 Univ Bretagne Sud, CNRS, UMR 6027, IRDL, 56100 Lorient, France; claire.dislaire@univ-ubs.fr (C.D.); yves.grohens@univ-ubs.fr (Y.G.); 2 Ecofeutre, Rue des Sports, ZA de Kerivan, 56550 Evellys, France; marion.muzy@ecofeutre.com

**Keywords:** softwood fiber, hardwood fiber, kraft pulping process, recycled fibers, molded pulp product, pulping process, mechanical properties, hygroscopic properties

## Abstract

With an increasing interest for molded pulp product (MPP) in the industry, it is important to fully understand how the manufacturing process is different from papermaking. One specific way to differentiate the processes is to compare their resulting products. As the paper industry uses several wood fibers with various pulping processes, it is interesting to compare some of these fibers, to further progress our understanding of the MPP process. In this study, six different wood fibers were used (as received) and analyzed to obtain the sample with the lowest moisture uptake and highest tensile properties. Scanning electron microscopy (SEM), Fourier transform infrared spectroscopy (FTIR), and fiber analysis module (MorFi) observations were performed, as well as moisture uptake measurements after sorption and tensile tests. We observed significant differences between the fibers tested. Kraft fibers (bleached softwood kraft pulp (BSKP), bleached hardwood kraft pulp (BHKP), and unbleached softwood kraft pulp (USKP)) showed smoother surfaces and less non-cellulosic molecules, such as hemicellulose, lignin, and pectin, in the SEM images. Bleached chemi-thermomechanial pulp (BCTMP) and recycled pulps (R-NPM and R-CBB) both showed non-cellulosic molecules and rougher surfaces. These results were confirmed with the FTIR analysis. With kraft fibers, MPP mechanical properties were lower than non-kraft fibers. Resulting moisture uptake is in between the recycled fibers (lowest moisture uptake) and BCTMP (highest moisture uptake). The removal of non-cellulosic molecules reduces the mechanical properties of the resulting MPP. The incorporation of non-wood molecules, as found in recycled fibers, also reduces the mechanical properties, as well as moisture uptake, when compared with BCTMP.

## 1. Introduction

In the last decade, the environmental impact of single-use plastic packaging has been studied to be reduced and their recycling was greatly improved. However, with the raw material being petroleum-based, industrials searched for a new packaging to produce with a bio-based and ideally biodegradable and/or compostable raw material. As wood cellulose fiber is a biobased material widely used by the pulp and paper industry, it has been thoroughly studied to replace petroleum-based products. Wood fibers’ properties were analyzed, paper machines were gradually improved [1], and several pulping processes were created to obtain paper with specific properties, such as higher mechanical properties, better brightness, or softness, depending on the product’s final application.

One of the first fiber extraction method used to make paper was a mechanical process called stone groundwood (SGW) and was first produced in the 1840s [1]. This method had a yield higher than 95%, but the resulting product had low strength, due to the wide distribution in fibers’ length, a high number of broken fibers, and higher fine content [2]. Further studies were performed to improve the pulp’s strength [3]. Shortly after, the refiner mechanical pulp (RMP) was developed, with the use of a refiner to disintegrate chips into individualized fibers. In this process, steam is produced, which softens the chips and helps maintain the initial fiber’s properties, when compared to SGW [1]. The thermomechanical pulp (TMP) was later developed to make a pulp with a higher strength, with the help of pressurized refiners to soften the chips. This technique improved the fibers’ macro-fibrillation, maintained longer fibers, and produced less fines, thus resulting in obtaining a pulp with higher strength [1]. The use of a mechanical pulp with wood fibers increased further in the 1870’s, when a modification was made to the process. A steam pretreatment was added to soften the lignin in the wood and improve the paper properties [4].

As the mechanical pulp maintains all wood molecules, it is hardly compatible for paper with a higher brightness and stability over time. Chemicals began to be introduced to the pulp, in order to remove the lignin molecules [5] and further improve their properties. Soda pulping was the first method developed [4,6]. It uses sodium hydroxide (NaOH) to cook the pulp and separate lignin and extractives from the pulp. In 1857, the sulfite process was developed, and bisulphite ions (H_2_SO_3_^−^) were used to remove lignin from the wood [4,7]. Another chemical pulping process was developed in 1879, kraft pulping, which used sodium hydroxide (NaOH) and sodium sulfide (Na_2_S) to delignify the pulp [5]. This cooking process is also called sulphate pulping.

To further improve mechanical pulps, industrials added chemicals at low concentration to improve the pulp’s strength, while limiting the pulp yield decrease. The chemi-thermomechanical pulp (CTMP) consists of a chemical pretreatment with a low amount of Na_2_SO_3_ or NaOH at lower temperature and pressure than chemical processes [1]. The pretreated chips are then refined following the TMP process.

With all these pulping processes, it is then possible to further bleach pulps to obtain a higher brightness, depending on the desired application. In mechanical pulps, the bleaching process reduces the pulp yield, as it removes the lignin, the molecule responsible for the paper yellowing, also called color reversion [1,4]. The bleaching of mechanical pulps is limited as the highest yield is looked for. The lignin is maintained but chromophores, the molecules responsible for the lignin color, are removed to maintain the highest yield pulp possible, while gaining brightness in the resulting pulp. To bleach mechanical pulps, sodium hydrosulfite (Na_2_S_2_O_4_) or hydrogen peroxide (H_2_O_2_) are used. After the bleaching process, bleached CTMP (BCTMP) is obtained.

For chemical pulps, the bleaching process is different and generally consists of several steps to efficiently remove lignin molecules and obtain a pulp with a high brightness [5,8]. Depending on the desired brightness, the bleaching steps may be modified. Some chemical agents used are oxygen, chlorine (Cl_2_), chlorine dioxide (ClO_2_), sodium hydroxide (NaOH), ozone, and others [8].

These processed pulps are widely used to produce paper and cardboard and to make 3D products called molded cellulose, molded fibers, or molded pulp products (MPP). The first patent describing the molded pulp products was filed in 1890 [9] and the first patent for a MPP manufacturing machine was filed in 1903 by Mr. Martin L. Keyes [10]. At first, its use in the industry was mostly for the production of egg trays, before interest grew in the last few years [11,12,13]. The MPP process is divided into four categories, fully described by the International Molded Fiber Association (IMFA) [14]. The method used in this study was the “thermoformed” or “thin wall” category. It uses more than two molds (usually four molds) in the shape of the finished product to obtain a product with a thinner, denser, and smoother surface than other categories that use one or two molds.

The pulp and paper industry have performed much research on the effects of fiber morphology on paper properties [15,16,17,18]. It was found that a higher paper density, obtained with process modification, improves the paper’s Young modulus [16]. Much research was also performed on fibers to increase knowledge and understanding for the pulp and paper industry, such as the influence of the fiber’s length and width on the paper’s mechanical properties [19] or the effect of additives on the inter-fiber bonding and dry-strength of paper [20,21].

As the MPPs process is different from the paper process after the pulping step, it is interesting to analyze the effects of fiber morphology on the properties of a product made with the MPP process. As MPP has drawn industrial attention in recent years, only few studies were made with this process and they focus on the process specificities and differences from papermaking [13,22,23]. To know which type of fibers were best suited to be used in the MPP process, six different fibers were compared in this study, in terms of morphological, hygroscopic, and mechanical properties.

SEM, MorFi analysis, and FTIR spectroscopy were performed to observe the differences between the fibers, depending on the pulping process (CTMP or kraft), the bleaching process, and whether the fibers were virgin or recycled. As cellulosic materials are naturally hydrophilic, moisture uptake analysis was done to the resulting MPP in five different water activities (a_w_). The Guggenheim-Anderson-de Boer (GAB) model was used to better understand the influence of fiber’s morphology on the resulting hygroscopic properties of each fiber tested. Tensile tests were done for the initial condition, as well as after the hygroscopic pretreatment, to better understand the influence of each fiber type on the resulting mechanical properties.

With this study’s results, we were able to further understand the influence of the fiber type used to produce MPP on the resulting hygroscopic and mechanical properties. As the MPP process is not fully understood, in terms of the parameters influence on the resulting product’s properties, more research will be done in the future to better understand the specificity of this process. It is important to know how wood fibers impact the resulting properties of the product made. With this information, we will be able to determine which fiber is to be used, depending on the application needed.

## 2. Materials and Methods

### 2.1. Materials Used and Production of MPP

To efficiently compare different wood fibers’ origins, six types of wood fibers were used and are described in Table 1. All fibers tested in this study were used without any further modification. The fiber’s lengths, described in the table, were obtained using MorFi analysis (MorFi Neo, TechPap, France), further described in 2.2.1. The Schopper-Riegler degree (Model SR/P, Regmed, Brazil) of all samples was obtained following European standard EN NF ISO 5267‒1 [24] and was used to obtain the drainability of the pulp. It is an important parameter to retrieve from the pulp in the paper industry, in order to efficiently remove water and dry the pulp using the appropriate process to use the least amount of energy as possible. The MPP test samples preparation was done in 3 steps for each wood fiber tested:

1. Manufacturing of 3D objects using the molded pulp product (MPP) process.
2. Cutting of samples in shouldered bars shape using a punch cutting device.
3. Sample conditioning, following the test procedure to be done.

**Figure 1:**
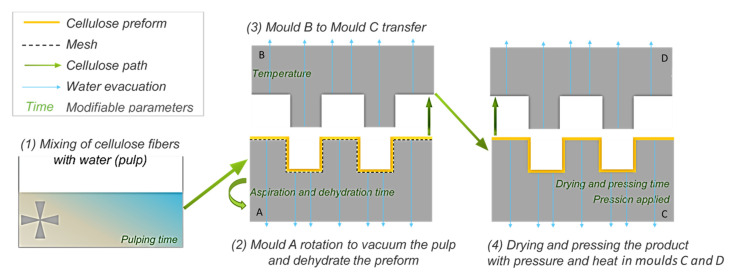
Diagram of the molded pulp product process used in this study, modified from Dislaire et al. [25].

For the first step, a molded pulp processing machine was used and described in Figure 1. It has been previously explained and studied by Dislaire et al. [25]. In the MPP process, several parameters can be modified to optimize the cycle time and energy consumption, as well as the structure and properties of the finished product.

For all fibers used in this project, the MPP process parameters were kept unchanged, in order to properly compare the effects of each type of fiber on the properties of the resulting MPP, with as few modifications on the process parameters as possible. As a result, the process parameters used for all MPPs made are as described in Table 2 with an indication of where each step was performed in Figure 1. The press closing time used in this study corresponds to a position where the moulds C and D are 1 cm apart but not in contact and pressing together yet. This short time is used to slow the mould’s speed approach before closing for the pressing step. The product’s drying step is done during the pressing time, when moulds C and D are in contact and pressing at a high temperature.

To efficiently compare the resulting MPPs made, it is important to obtain the same weight on the finished product. Thus, the aspiration time on mould A was modified for each pulp to produce food trays of about 25 g for all fibers analyzed. This variation of aspiration time is due to the drainability, given by Schopper Riegler degree (°SR). The drainability of each pulp changes the mold’s capacity to vacuum the pulp. A pulp with a higher °SR will result in a longer aspiration time, while a pulp with a lower °SR will have a shorter aspiration time to obtain a MPP with the same weight.

### 2.2. Description of the Characterisation Techniques of Molded Pulp Products Used

#### 2.2.1. Morphology Analysis Techniques Used

**Figure 2:**
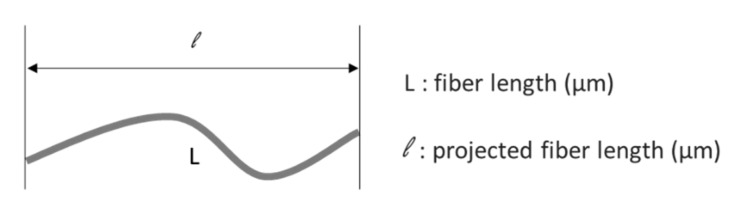
Scheme of how the fiber curl index (CI) is measured.

The first technique used was a fiber analysis module using a MorFi machine (MorFi Neo, TechPap, France). This method uses fiber pulp with a very high dilution of about 0.4 g/L to efficiently disperse the fibers and analyze each fiber and element. The pulp is screened through a high-speed camera and all elements seen are analyzed by the software included with the machine. The analysis lasts until 5000 fibers have been counted by the MorFi software. This technique allows us to obtain the following average fiber morphological parameters:

- Fiber’s length and width.
- Fiber’s macro-fibrillation (fibrils amount at the fiber’s surface).
- Curl index (CI) defined as shown in Equation (1) and Figure 2.
- Kinked fiber content and broken fiber content.
- Fines’ content (particles with a length of 200 µm or less).

To obtain further information about the fiber’s morphology, Scanning Electron Microscopy (SEM) observations were performed on the samples with a JSM-IT500HR microscope (Jeol Ltd., Japan), as well as Attenuated Total Reflection Fourier Transform Infrared spectrometer (ATR-FTIR Vertex70v, Bruker, MA, USA), to analyze the MPP samples for all fibers tested. All spectra were normalized at a 1028 cm^−1^ peaks to compare the resulting peaks.

(1) $$CI=\frac{L}{l}-1$$

#### 2.2.2. Hygroscopic and Mechanical Properties Analysis Techniques Used

**Figure 3:**
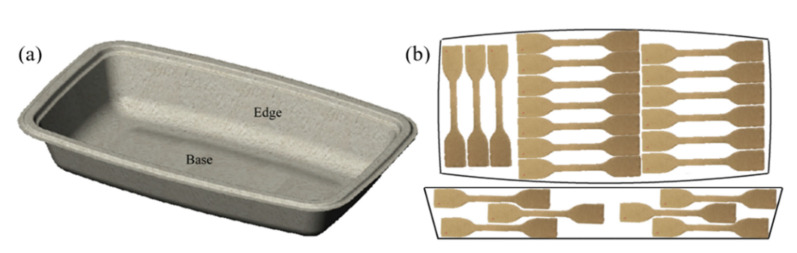
(**a**) A 3D model of the food tray used and (**b**) the sample cutting zones and dimensions on the food tray [25].

For the tensile tests, an MTS Synergie 1000RT (MTS Systems Corporation, machine was used with a load cell of 250 N and a speed maintained at 1 mm/min. For each pulp analyzed, a minimum of 5 samples were done. Testing samples were obtained by using a shouldered testing bar shaped punch cutter, with dimensions given in NF EN ISO 527-2 via a specimen 5 A type [26], to cut samples from the MPP. Samples were extracted as shown in Figure 3, with the same shape for all tests, thus reducing the possible errors and differences in length and width measurements. As thickness could be different, depending on the fiber tested, it was measured before testing for all samples. The average thickness and density of the samples tested are available in Table 3.

Initial tensile tests were performed on samples maintained at 23 °C (±2 °C), with a relative humidity of 50%. The sample’s Young modulus, also called elastic modulus, was obtained from the curve σ = f(ε) and using Equation (2), with Δσ as the sample’s stress difference in the elastic region of the tensile curve σ = f(ε) and Δε as the sample’s elongation difference in the elastic region. 

For the sorption analysis, desiccators at a specific water activity (a_w_) with saturated salts and at 23 °C (±2°C) were used and shown in Table 4. Water activity corresponds to the relative humidity (%hr) divided by 100. Saturated salts are used to maintain a specific water activity in the desiccator and each saturated salt allows us to obtain a water activity, as given in the Table 4. Samples were weighed three times before tensile tests were done. First, the samples were weighed before drying. Then, they were oven-dried at 105 °C for 48 h and weighed again to obtain their dried weight. Sorption analysis was performed, and samples were kept in controlled humidity chambers until equilibrium was reached. One last weighting was performed to have the samples weight after sorption analysis and tensile tests were performed in a climatic chamber.

(2) $$E\;\left(\mathrm{MPa}\right)=\frac{\Delta \sigma }{\Delta \varepsilon }$$

The following, Equation (3), was used to calculate the samples’ moisture uptake:

(3) $$\tau \;\left(\%\right)=\frac{W_{f}-W_{0}}{W_{0}}\;\times \;100$$

where *W_f_* is the sample’s weight after sorption analysis and *W*_0_ is the dried sample’s weight.

To have a better understanding of the tested fibers’ sorption behavior, the GAB model was used, and its equation is shown in Equation (4). τ is the final sample’s weight after the sorption test (moisture uptake), a_w_ is the desiccator’s water activity, and τ_m_ is the sample’s water content that was absorbed on the fiber’s surface as the first molecular layer, also called monolayer moisture content. C is an energy constant related to the difference in the water’s chemical potential (free enthalpy) between the monolayer and the upper layer (water molecules in pure liquid state) and is dependent of the water activity, as seen in Equation (6) [27]. As for K, it is an energy constant directly related to the sample’s multilayers’ heat properties. K defines the difference between the free enthalpy of the upper layer and the free enthalpy of the layers that are between the monolayer and the upper layer, starting from the second layer adsorbed [28].

(4) $$\tau \;\left(\%\right)=\frac{K\cdot \tau_{m}\cdot C\cdot a_{w}}{\left(1-\;K\cdot a_{w}\right)\times \left(1+K\cdot a_{w}\times \left(C\;-1\right)\right)}$$

(5) $$x_{1}=\frac{d^{\prime }}{K^{2}\times a^{\prime }}$$

(6) $$\frac{1}{C}=\frac{1}{C_{0}}\left(1+x_{1}\cdot a_{w}+x_{2}\cdot a_{w}^{2}+\ldots \right)$$

**Figure 4:**
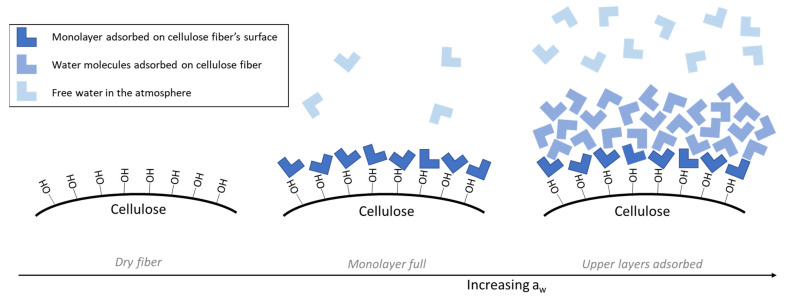
Fiber’s water uptake scheme on the surface of a dry fiber with increasing water activity.

A scheme of the monolayer addition to dry fiber and upper layers at higher water activities is shown in Figure 4. The monolayer content corresponds to the water uptake, at which all available bonding sites on the fiber’s surface are bonded with water molecules. With an increasing a_w_, water molecules will bond to the monolayer and so on to the upper layer, as further described and studied by Samyn [29]. The upper layer is the last layer of water molecules adsorbed on the fiber’s surface.

**Figure 5:**
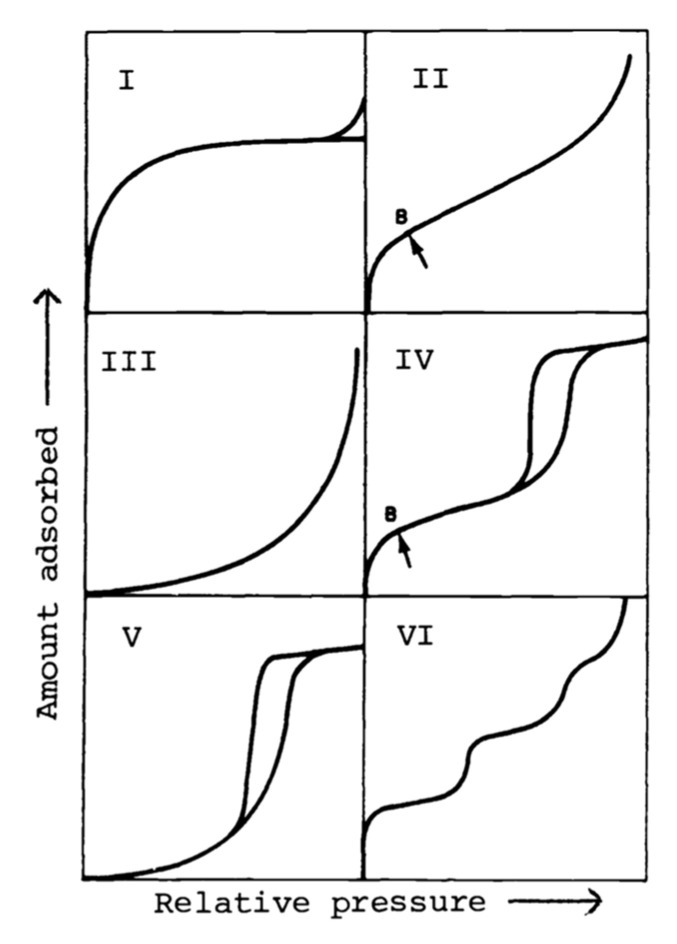
Adsorption isotherm types, as described by IUPAC [30].

IUPAC distinguishes the sorption isotherms into 6 types, as described in Figure 5 [30]. The type II isotherm is the most seen in porous materials and describes the type given in this study. The resulting GAB variables, for all MPP samples studied, are given in Table 5. The obtention of the variables for each sample was fully described by Blahovec and Yanniotis [27], with case A used for this study. The x_1_ variable, whose formula is given with Equation (5), was used in Blahovec and Yanniotis’ study [27]. Constants a’, d’, and R^2^ were obtained by the regression analysis of the curve a_w_ = f (a_w_/τ) from the method described by Blahovec and Yanniotis [27]. With the obtention of x_1_ and x_2_ equal to 0, the C value is obtained, which is dependent on the water activity, as given by Equation (6). With x_1_ at 0, it gives us the initial GAB model with C equal to C_0_. When all Gab variables are calculated, we are then able to use the GAB model Equation (4), by modifying the water activity (a_w_) from 0 to 1, to obtain the sorption curve on all the a_w_ range.

## 3. Results

### 3.1. MorFi Analysis

An efficient technique used to study the fibers morphology is the use of a fiber analysis module (MorFi). It allowed us to obtain information about the fibers’ average length and width for each pulp analyzed, as seen in Table 6.

The first interesting information given is the fiber’s length (L) and width (w) difference between hardwood (L = 711 µm and w = 18.6 µm) and softwood (L = 1286 µm and w = 27.4 µm), using the same pulping process (bleached kraft). There is also a difference in fiber’s morphology, whether the softwood kraft pulp was bleached (L = 1286 µm and w = 27.4 µm) or left unbleached (L = 1375 µm and w = 26.7 µm). However, this difference in width is minimal when considering standard deviation, with a width difference of 0.20 µm obtained. Both recycled fibers, as well as BCTMP fibers, have shorter fibers than all kraft fibers observed but have larger fibers than hardwood kraft fibers. As a result, the length/width ratios of recycled and BCTMP fibers are lower than kraft fibers.

### 3.2. SEM Observations

**Figure 6:**
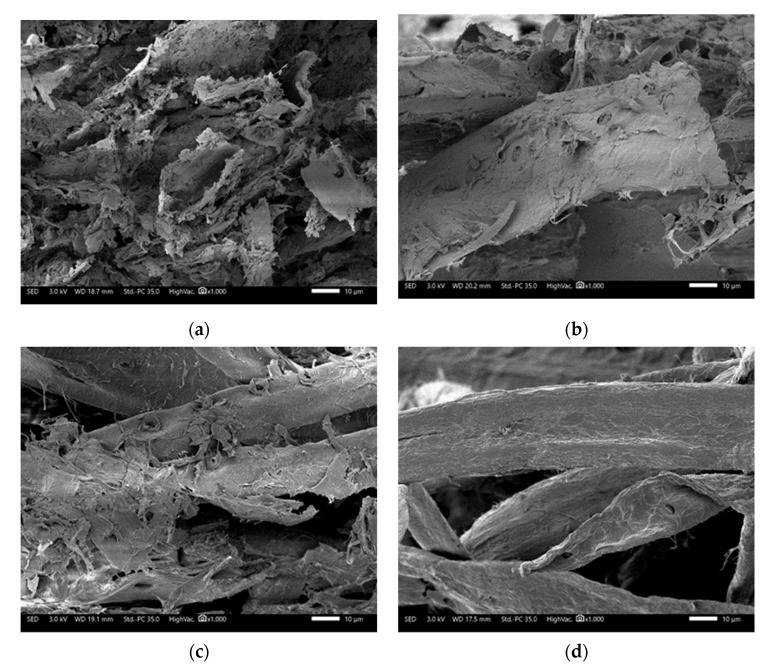

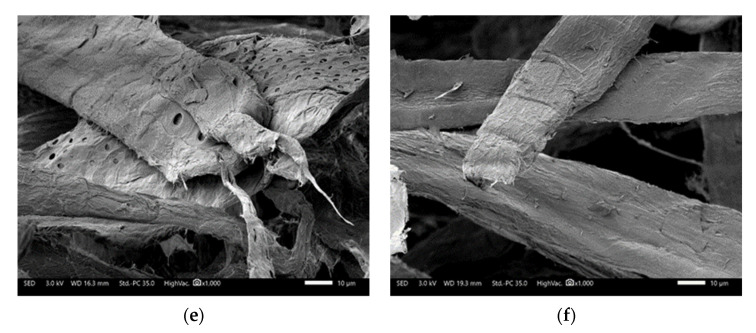
SEM images of fibers analyzed (scale bar is 10 µm for all images); (**a**) R-NPM, (**b**) R-CBB, (**c**) BCTMP, (**d**) BSKP, (**e**) BHKP, and (**f**) USKP.

To better understand the differences between the fibers tested, SEM analysis was performed to observe the morphology of the fibers surface (Figure 6). We observed that R-NPM Figure 6a fiber surfaces are highly obstructed from the previously added additives, such as ink, glue, and minerals. These fibers are also shredded from the past pulping processes, which may have occurred several times to the fibers.

R-CBB Figure 6b showed lower surface obstruction, as the raw material’s past purpose was for direct food contact, meaning that these fibers underwent less recycling processes. We also observed less shredding to the fibers’ surface and ending, when compared to R-NPM fibers.

With SEM analysis of BCTMP Figure 6c pulp, we observed the presence of elements on the fibers’ surface. These elements are non-cellulosic molecules, such as lignin, pectin, and hemicellulose, apart from cellulose preserved from the BCTMP process. It maintains most of molecules initially present in the wood in the final pulp, making it a high yield pulp. These molecules cover the fibers surface and are linked to the fibers.

All three kraft fibers given in Figure 6d–f show similar fiber morphology, with a smoother surface, when compared to recycled and mechanical pulps analyzed Figure 6a–c. It is interesting to note that a very low amount of wood molecules, such as lignin, pectin, and hemicellulose, were observed. The kraft pulping process was used to eliminate most of wood molecules, in order to only keep cellulose fibers in the final pulp, thus having a low yield.

### 3.3. FTIR Analysis

**Figure 7:**
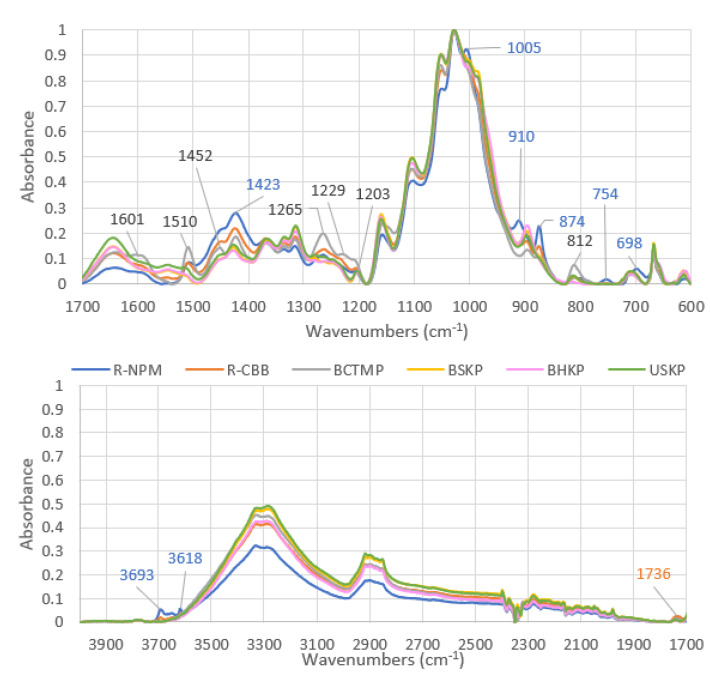
FTIR spectra of MPP samples analyzed. Specific peaks analyzed are given in colors to distinguish them with R-NPM (blue), R-CBB (orange), and BCTMP (grey).

To further compare the fibers used in this study, they were analyzed by FTIR spectroscopy. The resulting spectra, shown in Figure 7, were cut at 1700 cm^−1^, in order to have a better visualization of the peaks observed.

First, we observed that all kraft fibers (BSKP, BHKP, and USKP) showed very similar spectra, with peaks obtained at the same wavenumbers. As they are samples of cellulose fibers, with almost exclusively cellulosic molecules, as most non-cellulosic were removed, they were used in this study as a reference. It allows for the highlighting of the specific peaks that were also seen in the recycled and mechanical samples. These extra peaks are also shown in Figure 7 and, as each sample gave specific peaks, a color indication is given in relation to the graph legend.

The recycled cardboard box (R-CBB) sample had a few peaks different from the kraft fibers, but only one peak was the strongest of all samples analyzed, at 1736 cm^−1^. The other peaks were also observed on R-NPM and/or BCTMP, as 3693 cm^−1^, 1510 cm^−1^, 1423 cm^−1^, 1229 cm^−1^, and 874 cm^−1^. The peak at 1736 cm^−1^ corresponds to C = O, stretching in unconjugated ketone, carbonyl, and aliphatic groups xylan, which may correspond to agents added in previous uses of the paper [31].

With the analysis of the recycled newspapers and magazines (R-NPM) sample, peaks at 3693 cm^−1^ and 3618 cm^−1^ were obtained. They are known to be specific to kaolinite [32]. When analyzing the peaks, at 1423 cm^−1^, 1005 cm^−1^, 910 cm^−1^, 874 cm^−1^, 754 cm^−1^, and 698 cm^−1^, they were also characterizing mineral molecules, mainly from carbonate anion (sodium carbonate, calcium carbonate, and zinc carbonate) or the vibration of SI-O and Al-OH molecules [33]; these molecules are used in inks. Then, the peak at 1423 cm^−1^ can be attributed to aromatic skeletal vibration and CH in-plane deformation for lignin and hemicellulose [34].

The bleached chemi-thermomechanical pulp (BCTMP) mostly showed bands that are characteristics of non-cellulosic molecules, such as lignin and hemicellulose, with peaks at 1601 cm^−1^, 1510 cm^−1^, 1452 cm^−1^, 1265 cm^−1^, 1229 cm^−1^, 1203 cm^−1^, and 812 cm^−1^ [35,36,37].

The results, given by FTIR analysis, confirmed the SEM images analysis. Non-cellulosic molecules were present at the surface of cellulose fibers for recycled and mechanical samples, as opposed to kraft fibers.

### 3.4. Initial Mechanical Properties

**Figure 8:**
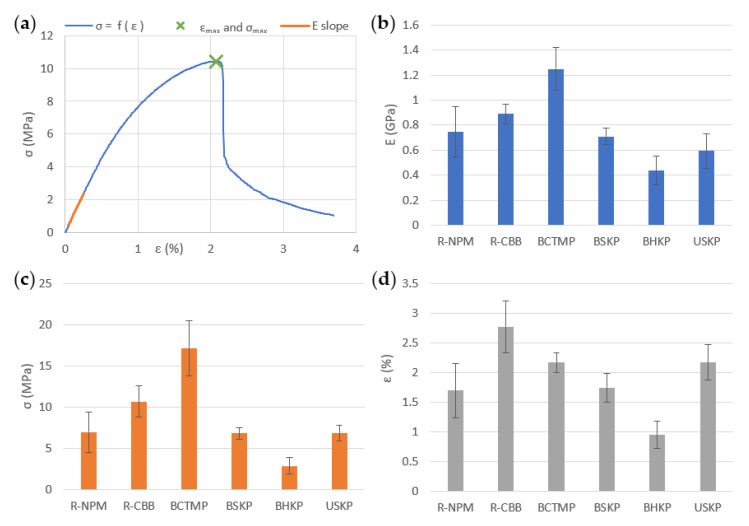
Mechanical properties in initial condition, depending on the fiber type used: (**a**) is a typical curve used to obtain the tensile results with the E slope to calculate, (**b**) the Young modulus (E) in GPa, (**c**) the stress at break in MPa, and (**d**) the strain at break.

For the initial mechanical properties, as seen in Figure 8, with about 400 to 700 MPa, the Young modulus of kraft fibers was lower than recycled fiber MPPs that have a Young modulus of about 800 MPa. BCTMP shows the highest mechanical properties with 1400 MPa, more than twice the kraft results.

Hardwood fibers have the lowest Young modulus, as they obtained the lowest stress and strain at break of all fibers tested. The Young modulus of all MPPs tested gave the same tendency as the stress at break, shown in Figure 8. The strain at break showed different results but with lower contrast between MPPs tested, as the highest variation was between R-CBB (with 2.77%) and BSKP (with 0.95%) of strain at break. All the other fibers were in the 1.7% to 2.2% range.

### 3.5. Molded Pulp Products Samples Moisture Uptake in Humid Environment and Resulting GAB Model

**Figure 9:**
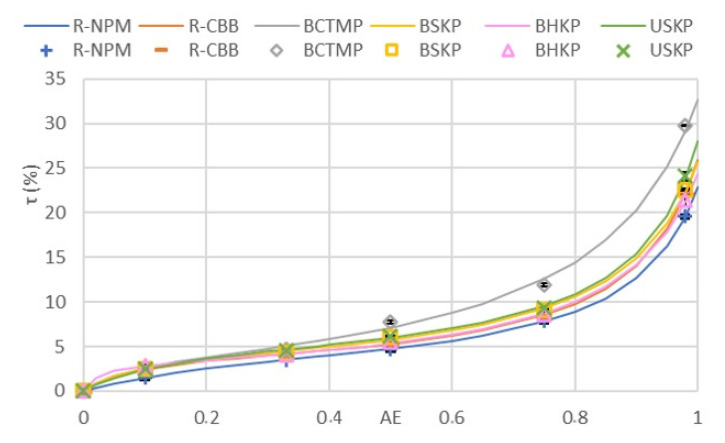
Moisture uptake of MPPs after sorption analysis.

The samples’ moisture uptake results, after adsorption analysis under different humid environments, are shown in Figure 9., as well as the GAB model variables for each fiber tested. The GAB curve efficiently follows the experimental results, as given by the R^2^ in Table 5. Of all samples tested, we observed the BCTMP as having a different curve, resulting in divergent GAB variables, when compared to the other samples’ variables. It is, in fact, the samples with the highest moisture uptake in all a_w_; all the other samples showed a lower and similar curve.

For BCTMP, the K value (0.84) was the lowest of all K values and τ_m_ was the highest, with 5.4%, while the τ_m_ of other samples were lower than 3%. As for C_0_ value, only R-NPM (9.21) was lower than BCTMP (11.2), whereas all other samples showed a higher C_0_ of up to 53.3 for BHKP. We can also observe that the recycled samples R-NPM and R-CBB have the lowest τ_m_ of all samples and the highest K value, with 0.90. The difference between the bleached and unbleached samples was observed, with a lower τ_m_ for unbleached kraft fibers, compared to the bleached kraft samples (BSKP and BHKP), as well as a higher K value of 0.90 for USKP and 0.88 for both BSKP and BHKP.

### 3.6. Mechanical Properties of Molded Pulp Product after Sorption Analysis

**Figure 10:**
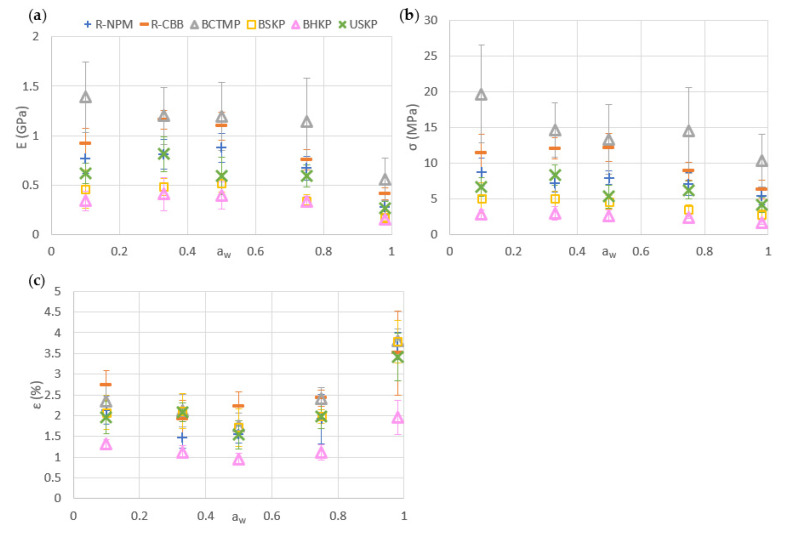
Mechanical properties after sorption analysis for fiber types; (**a**) Young modulus (E) in GPa, (**b**) Strength at break (σ) in MPa, and (**c**) strain at break (ε) in %.

The mechanical results, after water adsorption in a humid environment, are shown in Figure 10. We first observed that BCTMP has the highest Young modulus of all samples tested, for all the humidity ranges analyzed (water activity range from 0 to 1). Both recycled samples (R-NPM and R-CBB) show lower mechanical resistance than BCTMP; however, they have a higher mechanical resistance than all kraft samples. When looking at standard deviation, we observed that the R-CBB mechanical results were statistically close to the BCTMP samples, followed by the R-NPM and unbleached softwood kraft fibers (USKP). In kraft fiber’s samples, BHKP has the lowest Young modulus of all samples, whereas unbleached softwood fibers showed a higher mechanical resistance than bleached softwood fibers.

A decrease of Young modulus for all samples at higher a_w_ (0.98) was also observed. This mechanical variation was mainly observed in a strain at break (ε) increase at high a_w_, as shown in Figure 10c, whereas a slight decrease was observed in the strength at break at 0.98 a_w_ for all samples. A similar strain at break for all but the BHKP sample, for which it was lower, was observed.

## 4. Discussion

### 4.1. Influence of Fiber’s Origin and Morhplogy on Molded Pulp Products’ Initial Mechanical Properties

The first fiber’s origin comparison was between hardwood (BHKP) and softwood (BSKP) fibers that went through similar kraft and bleaching pulping process. It is widely known that hardwood fibers have shorter fibers than softwood [1,38,39,40]. These studies also showed that this difference modifies the resulting product’s mechanical properties, with lower strength at break for shorter fibers.

Fišerová et al. [41] studied the effect of bleaching on kraft pulps and observed that softwood has a higher tensile index than hardwood, whether they are bleached or left unbleached. As for the bleaching process, it showed that it reduces the mechanical properties, as unbleached pulp showed a higher tensile index than bleached pulp, for both hardwood and softwood pulps. In our study, we observed a higher strength at break for softwood fibers but a lower difference for the bleaching process of softwood kraft pulp. With fibers having similar length and width, the resulting initial strength at break is comparable, even with a bleaching process, but we obtained a higher strain at break for unbleached fibers.

The recycled fibers tested have shorter and thinner fibers than softwood kraft fibers and a similar morphology to hardwood kraft fibers. However, their mechanical properties are different from BHKP, with a higher Young modulus for both the recycled newspapers (R-NPM) and recycled cardboard box (R-CBB) samples. The recycling process reduces the mechanical properties of fibers, due to fiber’s morphological modifications, obtained during recycling, such as length reduction, hornification, or bonding alteration, due to non-wood additives on the fibers surface [42,43]. As shown with FTIR analysis, the recycled samples contain minerals such as kaolinite and carbonate-based clays. These molecules are often used in the paper industry to whiten and improve the brightness of the paper, but they may also decrease the paper strength, as it may disturb the bonding in between fibers [44]. It also seems like the recycled fibers are a mix between hardwood and softwood but also from kraft processes and mechanical processes, to obtain a higher Young modulus in this study than kraft fibers.

In other studies [45,46], CTMP products showed lower mechanical properties than kraft products. The fact that the MPP manufacturing process is different from papermaking also modifies the mechanical properties of the product made [11]. When comparing the fibers’ morphology, observed with SEM images and the mechanical results, we observed that having non-cellulosic molecules on the fibers’ surface (recycled and BCTMP) improved the Young modulus of the MPP. However, for fibers with a very low amount of non-cellulosic molecules on the surface (kraft fibers), we observed a lower tensile strength and Young modulus.

A similar difference may come from the recycled fibers with a chemical modification of the fibers, due to the presence of diverse molecules with specific properties, as detailed in several studies [47,48,49]. The presence of these molecules in the pulp changes the chemical behavior of the pulp, as well as the fiber’s surface morphology. These differences, thus, modify the mechanical properties of the resulting product, when compared to virgin fibers [50].

In this study, we observed that the absence of molecules, other than cellulose fibers, reduced the fiber’s resistance to a traction mechanism, as the inter-fiber cohesion is lower than for fibers having wood molecules on the surface to bond the fibers together. As high temperature and pressure were applied to MPP samples during production, non-cellulosic molecules, such as lignin and hemicellulose, observed a thermal modification. It is known that lignin has a glass transition at about 130 °C and further softens at 170 °C [36,51]. This softening of lignin in the pulp may help to form a higher cohesion between the fibers and the lignin.

Bleached chemi-thermomechanical pulp (BCTMP) samples have a higher Young modulus and strength at break than recycled fibers. It is possible to see that wood molecules, such as lignin and hemicellulose, as shown in FTIR analysis, further improve the product’s mechanical resistance, as the presence of these molecules allows a higher inter-fiber cohesion. Yet recycled fibers mainly have non-wood molecules and molecules that were added in previous paper-making processes, such as minerals (bentonite and titanium dioxide), ink, glue, and other paper additives, to obtain specific paper properties [49,52,53]. From the results obtained with these pulps, it seems that these synthetic molecules do not improve the inter-fiber cohesion, as they all have different and specific cohesion mechanisms.

### 4.2. The Effect of Fiber’s Morphology on Molded Pulp Products’ Moisture Uptake after Sorption Analysis

**Figure 11:**
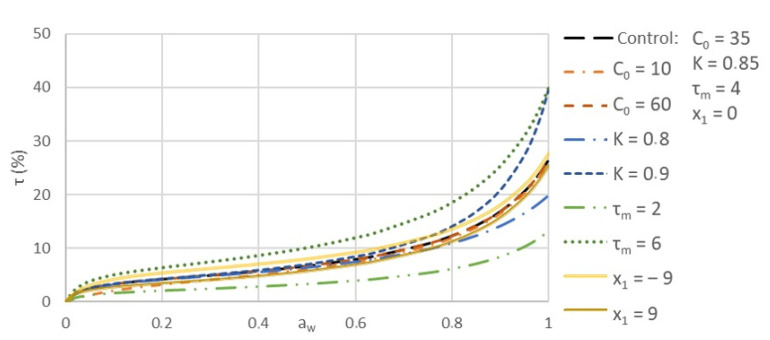
Influence of GAB constants on the resulting GAB curve.

To further understand the influence of K, C_0_, and τ_m_ values on the GAB curve, we compared a control curve with curves for which one variable was varied at a time. The result is shown in Figure 11. K and τ_m_ values both have a higher influence on the resulting GAB curve. Having a lower τ_m_ value allows the moisture uptake to be lowered for all a_w_ ranges. On the contrary, with a higher τ_m_ value, the moisture uptake is higher on all a_w_ ranges. Concerning the K constant, it mostly changes the curve at higher a_w_, beginning at 0.6. A lower K will decrease the curve at high a_w_, whereas a higher K will increase the water uptake at a higher a_w_. When comparing these values, we can also observe a higher moisture uptake on all a_w_ with τ_m_. This means that a higher moisture content in the monolayer has a much higher impact on the sample’s ability to adsorb water in humid environments, as compared to the multilayer’s heat properties (K value).

We also observed that C_0_ has a low influence on the GAB curve variation, even with a high difference in C_0_ value (±25), compared to K (±0.05) and τ_m_ (±2) used in this comparison analysis. A higher difference in the chemical potential between the monolayer and the upper layer, given by C_0_ value, has a very low impact on the material’s moisture uptake. As C constant is dependent on a_w_, given by Equation (5), it is easier to compare C_0_, as it defines C in dry conditions, where a_w_ is equal to 0.

With a negative x_1_, as obtained for all samples but BCTMP, the moisture uptake was reduced, whereas with a positive x_1_, the moisture uptake was increased. x_1_ was obtained with Equation (5). It is dependent on the K value. As BCTMP has the lowest K value (0.84) of all samples tested, this further increased x_1_ and, as a result, the sample’s moisture uptake.

Having the highest τ_m_ of all samples for BCTMP, as well as the lowest K value, explains the higher sample’s experimental moisture uptake. Hill et al. [54] analyzed the moisture uptake of natural fibers and observed that with a higher monolayer content, the resulting sample’s moisture uptake was also increased. The monolayer, on the fiber’s surface, is important to better understand the moisture behavior of the samples tested.

Zhang et al. [55] studied the effect of hemicellulose on the pulp moisture uptake and they observed that with a higher hemicellulose content, they obtained a higher moisture uptake. As hemicellulose molecules are hydrophilic, they enhance the water adsorption in the resulting pulp. In this study, the FTIR analysis showed that BCTMP contains hemicellulose, along with lignin. It seems that this presence greatly influences the sample’s moisture uptake, as opposed to kraft fibers or recycled fibers. Although hydrophobic lignin molecules are also in the BCTMP pulp, the highly hydrophilic nature of hemicellulose has a higher influence, translating in our study to a higher moisture uptake than the samples without hemicellulose and lignin.

### 4.3. Effect of Molded Pulp Products’ Moisture Uptake after Sorption Analysis on Samples’ Mechanical Properties

We observed that BCTMP had the highest mechanical properties, with a higher Young modulus for all a_w_ tested. When comparing these results with the initial mechanical properties obtained in Section 3.4, a sample with a higher Young modulus in initial conditions maintained a higher Young modulus after sorption analysis. With BHKP having the lowest Young modulus in initial conditions, we also obtained a lower Young Modulus after sorption analysis in all a_w_ tested.

The Young modulus decrease, observed at high a_w_, may be explained by the softening of the fibers of all samples. Salmén et al. [56] observed a softening effect of the wood fibers after adsorption of water molecules. They also showed the material’s softening is mainly induced by hemicellulose for a_w_ of 0.3 to 0.8. At higher a_w_, they showed that the amorphous phase of cellulose also softens the material. For BCTMP in our study, we obtained similar results with a higher water uptake, due to hemicellulose kept in the finished product, as opposed to the other fibers analyzed.

As humidity was increased, moisture uptake in the BCTMP also increased and, as a result, the tensile properties decreased, with lower Young modulus and stress at break but higher strain at break. An increasing moisture content in molded pulp product (MPP) samples may, as a result, create relaxation in the micro-compressions that may have been formed when the samples were dried during the process [57]. The relaxation could, in this case, allow the analyzed sample to have a higher strain at break. With this phenomenon, we can observe that as the strain at break increases, the stress at break decreases, thus decreasing the samples’ Young modulus. This further shows the fiber’s softening effect when a high moisture content is adsorbed by the sample.

It is also known that hornification, mainly observed in low yield pulps, such as kraft pulps, reduces the fiber’s swelling capacity and flexibility when in contact with water, thus resulting in a mechanical strength decrease [58,59]. This effect modifies the fiber’s surface morphology, with a reduction in their inter-fiber bonding capacity, due to fiber stiffening [60,61]. This phenomenon may explain the lower strength results in the kraft pulps (BSKP, BHKP, and USKP) analyzed in this study, when compared with the recycled (R-NPM and R-CBB) and mechanical (BCTMP) pulps.

For the mechanical softening of BCTMP, it is maintained up to 0.98 a_w_. This means that the mechanical decrease, observed for all MPP samples, was obtained due to the softening of amorphous cellulose at high a_w_.

**Figure 12:**
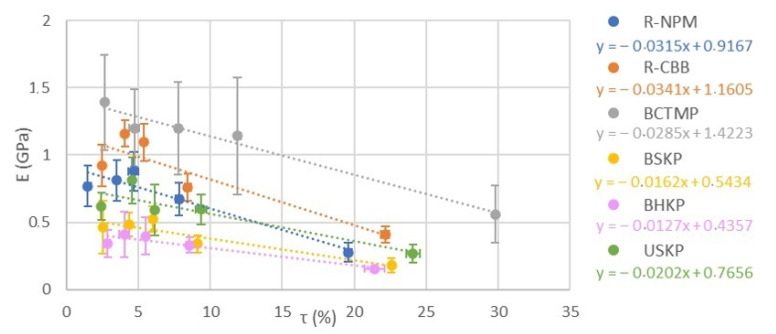
Moisture uptake influence on the Young modulus of all samples tested.

The curve in Figure 12 was used to further analyze the influence of moisture uptake on the mechanical properties of MPP samples. With this curve, we obtained an affine regression in the form “y = a·x + b”, with y as the Young modulus (in GPa), x as the water uptake, a as the curves’ slope giving information on the speed of change of E depending on a_w_, and b as the Young modulus in the sample’s dry state (E_dry_).

The theoretical samples of dry Young modulus obtained by the curve give us the same order as for Young modulus in the initial conditions (Section 3.4). The curve’s slope (a) differentiates the fibers used in two categories. The first one is for kraft fibers with a plot around −0.016 and the second is for BCTMP and recycled fibers with a plot around −0.031. This disparity may be explained by the morphological difference between the two categories. In kraft fibers, most of non-cellulosic molecules were removed (lignin, hemicellulose, and pectin), whereas most of wood molecules were maintained in BCTMP, and other types of molecules were added in recycled fibers, as shown in our FTIR analysis.

As there is a great potential variety of molecules that may be present in the recycled fibers, the effect of each of them on the resulting properties of the MPP samples will be complicated to precisely define. Several studies and reviews have performed research on the contaminants and particles in recycled paper and board [48,62] and found a high variety of both synthetic and natural molecules. Some of these molecules may increase the sample’s hydrophilicity, such a starch, whereas other molecules are added to reduce the paper’s hydrophilicity, such as waxes and wet strength agents. FTIR analysis showed us that clays, such as kaolin, were present in the recycles samples. These molecules may have an important role in the hygroscopic and mechanical properties of the resulting recycled samples (R-NPM and R-CBB). Naijian et al. [52] observed that the addition of kaolin in the paper reduced the water absorption. However, the addition of a high amount of kaolin also reduced the mechanical resistance of the paper. In our study, the presence of kaolin in the paper was beneficial to reduce the MPP sample’s moisture uptake. As the mechanical properties of the recycled samples were kept higher than kraft fibers, it seemed that the amount of kaolin in the pulp was sufficiently low to avoid a decrease in the mechanical resistance.

Having non-cellulosic molecules on the cellulose fibers surface, thus, allowed the MPP samples to have higher initial and dry Young modulus but also a higher Young modulus loss with increasing moisture uptake.

When comparing the molecules covering the fibers’ surface, it seems that wood molecules, naturally existing and maintained in BCTMP, allowed the resulting MPP to obtain a higher Young modulus on all conditions tested in this study. With recycled fibers, the origin and amount of molecules covering the cellulose fibers were complicated to analyze. Moreover, the bonding and hydrophilic properties between these molecules and the fiber surfaces, as well as other molecules, may be significantly different. Each recycled paper previously underwent specific chemical pretreatments and an addition of agents (molecules) to obtain the desired properties of the paper. These molecules were artificially added and mostly synthetic or minerals. Their cohesion to cellulose fibers, as well as all other additives that may be in contact, may be much lower than the cohesion between molecules that were originally in the wood, such as lignin and hemicellulose.

In our study, the MPP made with a pulp having non-cellulosic molecules on the cellulose fiber’s surface allowed the product to have the desired mechanical properties for packaging applications.

We also observed that, even with longer fibers, as given by morphological analysis, kraft fiber based MPPs showed lower mechanical properties. The impact of the fiber’s length was lower than the effect of preserving the wood molecules in the pulp or even adding molecules in the pulp (recycled fibers). However, when comparing the fiber’s origin, obtained with the same pulping process as used for BSKP and BHKP, we observed that using softwood fibers (BSKP) allowed the resulting MPP to gain a higher Young modulus, both in the initial conditions and after moisture uptake, when compared to hardwood fibers (BHKP).

The effect of bleaching was also observed with higher initial properties for USKP, compared to BSKP. A higher moisture uptake was observed for the unbleached fibers for all a_w_ tested, as compared to bleached fibers. It seems that the bleaching process, performed on chemical pulps, further removed non-cellulosic molecules that could have been left after the kraft process. Thus, it changed the properties of the resulting MPP made with an increase in hydrophilicity. With this difference between both fibers, MPP, made with USKP in this study, gave higher mechanical properties under humid conditions in all a_w_ tested than BSKP samples.

## 5. Conclusions

In this study, we compared 6 types of wood fibers to better understand the influence of fiber’s origin and pulping process on the moisture uptake and mechanical properties of molded pulp products (MPP). As it showed the lowest Young modulus in all conditions tested, the use of hardwood is discouraged to produce MPP with desired mechanical properties for packaging solutions. Additionally, the bleaching process used for bleached softwood kraft pulp (BSKP), as opposed to unbleached softwood kraft pulp (USKP), decreases the mechanical properties of the resulting MPP even with a lower moisture uptake obtained for MPP made with bleached fibers.

For MPPs made with recycled fibers, we obtained a lower moisture uptake than with BCTMP fibers; however, the resulting mechanical properties were also lower. MPPs made with kraft fibers had the lowest mechanical properties overall, suggesting us that the removal of all but cellulose molecules in wood fibers reduces the inter-fiber cohesion and is unfavorable to be used in MPP process. As mechanical pulp (BCTMP) contains non-cellulosic molecules, such as hemicellulose and lignin, a higher Young modulus and moisture uptake were obtained on MPP samples. As the MPP process uses high temperature and pressure on product with low thickness, lignin molecules reach their softening point, which may increase the chemical bonding in between the molecules when the product is cooling down.

Also, the presence of hemicellulose, a highly hydrophilic molecule, in BCTMP increased the sample’s moisture uptake to a higher content, when compared to the other samples tested. However, the mechanical properties of BCTMP after sorption is maintained higher than all other samples. This higher moisture uptake may have a role in the bonding between the molecules allowing a higher tensile resistance.

With the results given in this study, we observed that BCTMP-based MPP gave the most promising mechanical properties with the highest Young modulus and strength at break for all samples analyzed. Despite a higher moisture uptake in humid environment, MPPs made with BCTMP fibers maintained the highest mechanical properties, water adsorption playing a plasticizing role in higher a_w_.

## Figures and Tables

**Table 1:** Description of the fibers tested in the study.

Samples	Description of Fibers	Supplier	Wood Species Used	Fibers’ Mean Length (µm)	Schopper–Riegler Degree (°SR)
R-NPM	Recycled newspapers and magazines	Locally collected	Unknown	555	53
R-CBB	Recycled cardboard box	CBB wastes	Unknown	642	32
BCTMP	Bleached chemi-thermomechanical pulp	Rottneros	Spruce	615	27
BSKP	Bleached softwood kraft pulp	Fibre Excellence	Pine	1286	14
BHKP	Bleached hardwood kraft pulp	Fibre Excellence	Mix of hardwood	711	16
USKP	Unbleached softwood kraft pulp	Ahlstrom-Munksjö	Pine and spruce	1375	17

**Table 2:** Description of the MPP process parameters used for all fibers tested in this study.

	Step Number in Figure 1	Process Parameter
Pulp concentration	1	0.5%
Time of pulping	1	15 min
Dehydration time	2	15 s
Mould B temperature	3	120 °C
Mould C and D temperature	4	240 °C
Press closing time	4	0.1 s
Pressing time	4	30 s

**Table 3:** Average weight and density of MPP samples tested in this study.

Sample	Weight (g)	Density (g·cm^−3^)
R-NPM	0.64 (±0.05)	0.61 (±0.05)
R-CBB	0.68 (±0.09)	0.55 (±0.06)
BCTMP	0.65 (±0.06)	0.57 (±0.05)
BSKP	0.65 (±0.09)	0.53 (±0.02)
BHKP	0.65 (±0.09)	0.52 (±0.03)
USKP	0.64 (±0.1)	0.57 (±0.03)

**Table 4:** Saturated salts and their water activity used to test the sorption behavior of MPPs.

a_w_	0.10	0.33	0.50	0.75	0.98
Saturated salt	KOH	MgCl_2_	Climatic chamber	NaCl	K_2_SO_4_

**Table 5:** Value of all GAB variables obtained for each sample tested in this study.

Sample	K	C_0_	τ_m_	x_1_	R^2^
R-NPM	0.89 (±0.03)	9.69 (±2.99)	2.42 (±0.60)	−2.67 (±3.12)	0.9862 (±0.0012)
R-CBB	0.90 (±0.01)	27.57 (±14.07)	2.56 (±0.26)	−6.47 (±1.58)	0.9972 (±0.0020)
BCTMP	0.84 (±0.04)	12.83 (±2.01)	6.67 (±3.34)	10.60 (±15.12)	0.9843 (±0.0105)
BSKP	0.88 (±0.00)	19.14 (±0.79)	2.91 (±0.14)	−5.05 (±1.15)	0.9910 (±0.0034)
BHKP	0.88 (±0.01)	41.17 (±13.98)	2.90 (±0.24)	−3.24 (±4.86)	0.9931 (±0.0033)
USKP	0.90 (±0.01)	16.15 (±5.12)	2.73 (±0.35)	−6.42 (±2.75)	0.9948 (±0.0059)

**Table 6:** Fibers’ length and width, obtained by MorFi analysis.

Sample	Fiber’s Length (µm)	Fiber’s Width (µm)	Length/Width Ratio
R-NPM	555 (±10.7)	20.5 (±0.06)	27.05
R-CBB	642 (±20.7)	23.2 (±0.32)	27.63
BCTMP	634 (±24.3)	25.2 (±0.67)	25.13
BSKP	1286 (±27.5)	27.4 (±0.32)	46.89
BHKP	711 (±6.9)	18.6 (±0.15)	38.15
USKP	1375 (±24.5)	26.7 (±0.20)	51.49

## Data Availability

Not applicable.
